# Evaluation of remifentanil anesthesia for off-pump coronary artery bypass grafting surgery using heart rate variability

**DOI:** 10.3892/etm.2013.1108

**Published:** 2013-05-10

**Authors:** AIHUA SHU, LEYUN ZHAN, HAIBIN FANG, EN LV, XIAOBO CHEN, MINGYU ZHANG, QIANG WANG

**Affiliations:** Department of Anesthesiology, Three Gorges University People’s Hospital, Yichang, Hubei 443000, P.R. China

**Keywords:** heart rate variability, remifentanil, target-controlled infusion, off-pump coronary artery bypass grafting

## Abstract

Heart rate variability (HRV) was used in the present study to evaluate a target-controlled approach compared with a constant-rate infusion for remifentanil anesthesia during off-pump coronary artery bypass grafting (OP-CABG) surgery. A total of 65 patients with American Society of Anesthesiologists (ASA) physical status II or III, who were aged 60–85 years and scheduled for OP-CABG, were selected for the study. All patients were administered an intramuscular premedication of 10 mg morphine and 0.3 mg scopolamine. In group I, remifentanil was infused using a target-controlled approach at 1.5–5.0 ng/ml, and in group II, remifentanil was infused at a constant-rate of 0.05–1.0 *μ*g/kg/min and at additional single increments of 1 *μ*g/kg when appropriate. The heart rate and other hemodynamic monitoring indices of the patients, including the mean arterial pressure, central venous pressure, pulmonary artery pressure and pulmonary capillary wedge pressure, were monitored at various time points, including prior to induction (T_0_), at extubation (performed intraoperatively; T_7_) and at 24 h post-surgery. The HRV indices, including total power (TP), low frequency (LF) and the LF/high frequency (HF) ratio of power (LF/HF), were reduced following induction at T_0_ and remained low at 24 h post-surgery. At T_5_ (right coronary or left circumflex artery anastomosis) and T_7_ (tracheal extubation), all the HRV indices, with the exception of the HF power, were significantly increased (P<0.05). Additionally, the TP, LF and LF/HF values in group II were higher at T_5_ compared with those in group I (P<0.05). Remifentanil target-controlled infusion is superior to constant-rate infusion in suppressing the stress response during OP-CABG, maintaining the balance of the cardiac autonomic nervous system and promoting the recovery of the autonomic function following surgery.

## Introduction

Coronary artery bypass grafting (CABG) surgery, including on-pump CABG and off-pump CABG (OP-CABG) surgery, is one of the landmark procedures in the history of cardiac surgery and has saved millions of individuals afflicted by coronary artery disease. OP-CABG is the more recent of the two techniques, with the reported benefits of reductions in blood loss and neurological impairment (1). Moreover, the OP-CABG procedure avoids the adverse effects of extracorporeal circulation on the physiological functions of patients, thus reducing respiratory problems and resulting in fewer complications and a faster recovery. OP-CABG is also associated with reduced risk-adjusted mortality and morbidity (2,3). Since OP-CABG cases require vigilant anticipation of the surgical steps, the success of this technique relies upon skilled hemodynamic management, close communication with the cardiothoracic surgeon and controlled sedation and analgesia. Currently, remifentanil is widely used in cardiac surgery due to its sedative, analgesic and anti-tussive properties, which allow a prolonged infusion without drug accumulation (4). In addition, remifentanil target-controlled infusion (TCI) may be applied in OP-CABG for fast-track anesthesia, which combines a real-time pharmacokinetic model with an infusion pump and allows the administration and maintenance of a constant blood concentration of remifentanil. TCI also enables changes in the blood concentration to be made rapidly and easily. This procedure has a high safety and efficacy and is suitable for cardiac surgeries (5). In general, as there are no standardized methods for determining the state of analgesia, anesthesiologists determine the surgical stimulation intensity based on their clinical experience and adjust the depth of anesthesia accordingly (6). The lack of objective monitoring indices for immediate stress responses makes it difficult to control the quality of anesthesia. In clinical practice, individual signs of inadequate analgesia may include a change in hemodynamic indices, including the heart rate (HR) and mean arterial pressure (MAP), and a change in the concentration of plasma epinephrine (E), cortisol (COR), blood glucose (BG) and lactate (LAC), all of which indicate the intensity of the stress response to a certain extent (7,8,9). However, hemodynamic indices may be affected by a number of factors and thus differ significantly among individuals. The HR and MAP are classical parameters for registering the adequacy of analgesia, whereas the non-invasively measured MAP is not suitable for closed-loop control since it supplies the measured values in intervals of several minutes only. Similarly, blood biochemical parameters are more objective but it is not possible to monitor them conventionally in a real-time manner. The various parameters of heart rate variability (HRV) offer the possibility of supporting the autonomic response of the HR without the induction of a further monitor. HRV measures the variation in the HR and is calculated by analyzing the beat-to-beat intervals from an electrocardiogram (ECG) or an arterial pressure tracing. In this way, the parasympathetic and sympathetic activities of the autonomic nervous system (ANS) are visualized. Therefore, the non-invasive HRV analysis may quantitatively and rapidly evaluate the tension and balance of cardiac sympathetic and vagal nervous activities, as well as their effects on cardiovascular system activity (10). The overall levels of functioning and resilience, the degree of adaptability and the amount of progress that patients are making with therapy may be quantified using the HRV analysis, which has been widely applied in basic and clinical research studies (11–13). Thus, the continuous monitoring of post-operative HRV remains necessary. The present study aimed to investigate the changes in the HRV of patients undergoing OP-CABG with remifentanil TCI anesthesia and to assess the clinical importance of HRV as a real-time monitoring index of the stress reaction intensity.

## Patients and methods

### Patients

Patients with coronary heart disease (CHD) who exhibited stable angina pectoris at room temperature and took 25–100 mg metoprolol daily, with no other β-blockers or only metoprolol combined with a calcium antagonist, were selected for the present study. The patients underwent OP-CABG in the Three Gorges University People’s Hospital (Yichang, China) between June 2008 and May 2010. The exclusion criteria were as follows: i) a conventional ECG showing irregularities that interfered with the HRV analysis, including a left or right bundle branch block, left ventricular hypertrophy, atrial fibrillation or flutter, a second- or third-degree atrioventricular block or an R-wave to R-wave (RR) interval of >0.24 sec; ii) a pacing cardiac rhythm; iii) the presence of diabetes; iv) the presence of central nervous system (CNS) or ANS diseases; or v) a pre-operative use of CNS drugs. A total of 65 patients, consisting of 49 males and 16 females aged 68.4±10.2 years, with a mean body weight of 67.3±10.8 kg, were selected. Of these patients, 33 exhibited hypertension, two presented with aneurysms, 51 presented with ≥3 lesions, 11 with 2 lesions and three with a single lesion on the left main coronary artery. Furthermore, 38 patients demonstrated grade II pre-operative ventricular function and 27 demonstrated grade III pre-operative ventricular function. The left ventricular ejection fraction (LVEF) was >50% in 39 patients and 45–50% in 26 patients. The patients were randomized into a target-controlled group (group I) with 32 patients and a constant-rate infusion group (group II) with 33 patients. Approval for the present study was obtained from the medical ethics board of The Three Gorges University People’s Hospital. Prior to the surgery, the risks were fully explained and informed consent was obtained from each patient.

### Anesthesia method

All patients were injected with 10 mg morphine and 0.3 mg scopolamine prior to entering the operating room. The HRs and blood pressures of the patients were monitored continuously using an ECG and sphygmomanometer, and the pressure in the right radial artery was measured using a puncture catheter under local anesthesia. Intravenous anesthesia was induced in the two groups using the sequential administration of 10 mg dexamethasone, 0.05 mg/kg midazolam, 0.15 mg/kg etomidate, 0.15 mg/kg vecuronium and 10 *μ*g/kg fentanyl. Tracheal intubation was conducted 5 min later and TCIs of propofol were then performed to maintain the plasma concentration (Cp) at 1.0–2.5 *μ*g/ml and the bispectral index (BIS) at 45–60. Remifentanil was infused in a target-controlled manner at 1.5–5.0 ng/ml for group I and at a constant–rate of 0.05–1.0 *μ*g/kg/min for group II, with additional single increments of 1 *μ*g/kg remifentanil when appropriate. The central venous pressure (CVP), pulmonary artery pressure (PAP), pulmonary capillary wedge pressure (PCWP) and partial pressure of end-tidal CO_2_ were monitored and the arterial blood gas was intermittently checked. Following the opening of the central vein, 0.5–2.0 *μ*g/kg/min nitroglycerin, 1–3 *μ*g/kg/min dopamine and 10–80 ng/kg/min norepinephrine were continuously infused. Vecuronium was administered to maintain muscle relaxation. A single injection of 20–100 *μ*g phenylephrine was used to stabilize the blood pressure when changing the position of the patients and the parameters of the ventilator were adjusted to maintain the indicators of the arterial blood gas within the normal range. In groups I and II, following sternal closure, a single intravenous injection of 100 mg tramadol and 5 mg tropisetron and a controlled analgesia pump were used to manage any post-operative pain. The analgesia pump formula consisted of 1.0 g tramadol, 100 mg flurbiprofen and 0.9% sodium chloride in 100 ml, which was administered at a rate of 2 ml/h, controlled to 0.5 ml per infusion and then locked for 15 min.

### Intensive care unit (ICU) monitoring

Following surgery, the patients were sent to the ICU, where the ECG, arterial blood pressure (ABP), oxygen saturation (SpO_2_), CVP, PAP, PCWP, blood gases and electrolytes were monitored. Vasoactive drugs were infused to maintain hemodynamic stability and to support respiratory function. The monitoring was continued following tracheal extubation.

### Monitoring indices

The hemodynamic indices, including the HR, MAP, CVP, PAP and PCWP, were monitored and recorded prior to induction (T_0_), following induction (T_1_), during endotracheal intubation (T_2_), 2 min subsequent to the chest being opened (T_3_), at exposure and subsequent to fixing the anterior descending artery (T_4_), subsequent to exposing and fixing the right coronary and/or circumflex artery (T_5_), at the end of surgery (T_6_), at tracheal extubation (T_7_) and at 24 h post-surgery (T_8_). The total amounts of nitroglycerin, dopamine, norepinephrine, and phenylephrine that were used in the two groups during the surgery were also measured. Arterial blood was drawn to check the blood gas levels at T_0_, T_2_, T_3_, T_5_, T_6_, T_7_ and T_8_. Following centrifugation, the plasma concentrations of E, COR, BG, and LAC were measured. The corrected value was calculated as follows: Measured value x basal hemoglobin concentration/actual hemoglobin concentration. A HXD-I multi-function monitoring system (Huaxiang company, Harbin, China) was used to measure the HRV indices by frequency domain analysis, with the frequency range of the total power (TP) as 0–0.5 Hz. The low frequency (LF) was 0.03–0.15 Hz and the high frequency (HF) was 0.15–0.40 Hz. The LF/HF ratio of power (LF/HF) was calculated. These indicators were recorded at T_0_, T_2_, T_3_, T_5_, T_6_, T_7_ and T_8_.

### Statistical analysis

All the analyses were carried out using SPSS 13.0 software (SPSS, Inc., Chicago, IL, USA). The TP, HF and LF data were transformed by a natural logarithm, while the LF/HF was transformed by the square root. All data are presented as the mean ± standard deviation (SD). The Student’s t-test was used for the comparison of the measurement data, including the age and weight of the patients, the drug dosage and the surgery time, between the groups. The single-factor repeated measures analysis of variance was used for the comparison of the acquired HRV relevant indices at each time point for the two groups. Multiple comparisons were conducted using the least significant difference (LSD) method. The χ^2^ test was used to examine the count data. P<0.05 was considered to indicate a statistically significant difference.

## Results

### Patient characteristics

A total of 65 patients who underwent a CABG procedure between June 2008 and May 2010 were randomized into a target-controlled group (group I) consisting of 32 patients and a constant-rate infusion group (group II) consisting of 33 patients. During the perioperative period, no patient succumbed, had serious complications or required changing to extracorporeal circulation. The basic characteristics of the patients in the two groups, including gender, age, body weight, LVEF, duration of surgery, number of bypasses and remifentanil, metoprolol, nitroglycerin and esmolol dosages, showed no significant differences between the groups with the exception of the phenylephrine dosage. The patients in group II were administered greater amounts of phenylephrine than those in group I (P<0.05); the phenylephrine was mostly used when moving the heart and anastomosing the right coronary and/or circumflex artery vascular bridge (Table I).

### Changes in hemodynamic indices

Despite the similar characteristics observed in the two groups following the CABG procedure, changes in the hemodynamic indices, including the HR, MAP, CVP, PAP and PCWP, were analyzed during the perioperative period at various time points from T_0_ to T_8_ in the two groups. Among these parameters, a significant increase was observed in the HR and MAP, but only between the T_0_ and T_7_ time points for each group (P<0.05; Table II), whereas no significant changes occurred in CVP, PAP or PCWP among the various time points during the perioperative period.

### Plasma levels of E, COR, BG and LAC

Similarly, the levels of E and COR in groups I and II were significantly elevated at the T_7_ time point compared with those at T_0_ (P<0.05). The levels at the other time points, (T_1_, T_2_, T_3_, T_4_, T_5_, T_6_ and T_8_), were not significantly different from those at T_0_ (Table III), which was consistent with the changes in the HR and MAP observed during the perioperative period (Table II). The BG and LAC levels increased at T_3_, T_5_, T_6_, and T_7_ in the two groups. The levels in group II were significantly higher than those in group I at T_5_, T_6_ and T_7_ (P<0.05). However, the levels were restored to baseline at T_8_ (Table III).

### HRV changes in different time points

**Figure 1.**
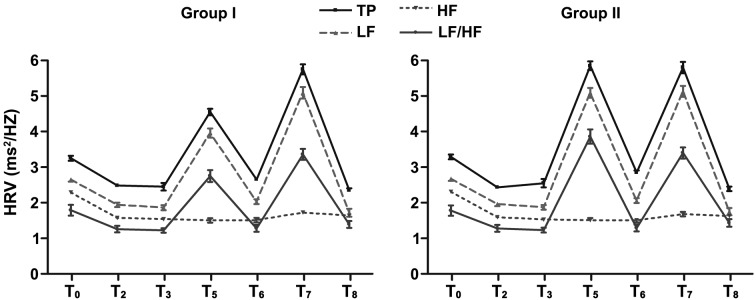
Changes in HRV in group I and group II. Data are presented as the mean ± SD, group I (n=32), group II (n=33). TP, total power; LF, low frequency; HF, high frequency; LF/HF, LF/HF power ratio; HRV, heart rate variability; SD, standard deviation.

In a previous study, the BIS, a well-validated measure of anesthetic depth, did not reflect the level of intraoperative stress (14). However, HRV may reflect ANS activity and stress-related sympathetic activation may induce a similar change in the observed HRV pattern (15). In the present study, the HRV indices, including the TP, LF, HF and the LF/HF, began to decrease following the induction of anesthesia at T_0._ These parameters were significantly reduced in each group at T_2_, T_3_, T_6_ and T_8_ when compared with those at T_0_ (P<0.05). The HRV indices were not restored until 24 h post-surgery. The values of TP, LF and LF/HF were significantly increased at T_5_ and T_7_ (P<0.05). The values of TP, LF and LF/HF in group II were significantly higher compared with those in group I at T_5_ (P<0.05). At other time points, there were no significant differences between the two groups (Table IV). Thus, the dynamic changes of the TP, LF, HF and LF/HF were observed in the two groups at each time point. The indices at T_5_ and T_7_ indicated peak values, which also showed significant differences between the groups (Fig 1).

## Discussion

Coronary heart disease (CHD) is currently the most common form of heart disease and an significant cause of premature mortality throughout the world. Patients with CHD have a lower vagal tone, increased sympathetic activity and reduced cardiovascular response to stress and adaptive regulation. These are significant factors that may trigger ventricular arrhythmia, myocardial infarction, abnormal changes in heart function and other cardiac emergencies (15,16). Effective prevention and therapy for CHD pose a major challenge to the entire medical community. The perioperative measurement of HRV is a relatively new method of assessing the balance of the ANS (17), which refers to successive small differences between the cardiac cycles resulting from the cardiac autonomic regulation of the sinus node. The TP (0–0.5 Hz) reflects the general autonomic tone. The LF (0.03–0.15 Hz) is regulated by the sympathetic and vagal nerves, with the sympathetic nerve having the dominant role. The HF (0.15–0.40 Hz) is associated with respiratory rhythm, reflecting the vagal tone, and the LF/HF represents the balance between the sympathetic and vagal tone (18). A reduced HRV is an index of sympathetic nerve predominance, including the parasympathetic nerve withdrawal state, in the ANS. Moreover, a reduced HRV has been observed to predict certain cardiovascular events, including sudden death and myocardial infarction, in patients with coronary artery disease or in apparently healthy subjects (19,20). However, the pathophysiological link between a reduced HRV and CHD is not well understood. Dekker *et al* observed that a reduced HRV was the strongest independent predictor of focal coronary atherosclerosis in patients who had undergone a prior coronary artery bypass surgery (21). Similarly, the present study demonstrated that the HRV indices, including the TP, LF, HF and LF/HF, began to decrease following the induction of anesthesia at T_0_ and were not restored until 24 h post-surgery, suggesting that a reduced HRV may be a good predictor of pathological changes in patients following OP-CABG. Therefore, considering the patient’s age, cardiac function, history of myocardial infarction and other relevant factors, a reduced HRV is an independent factor predicting sudden cardiac death and clinical risk (22). Dupliakov *et al* (23) confirmed that a change in the frequency domain of HRV was also associated with complications and the prognosis. Certain β-receptor blockers, including metoprolol, are often used to improve the LF/HF in such patients. Therefore, monitoring the changes in HRV in patients with coronary artery disease is crucial for reducing the incidence of adverse events during the perioperative period (24–26).

A previous study showed that surgical stress provoked the hypothalamic activation of the sympathetic ANS and that HRV reflected sympathetic activation during orthostatic and mental stress (27). HRV is affected by anesthesia, and various anesthesia methods and drugs have differing effects (28,29). Sato *et al* (30) described a reduced LF/HF attributable to a reduction in LF in patients with sevoflurane or propofol anesthesia. It was concluded that the choice of the anesthetic did not appear to play a critical role in HRV. By contrast, Kanaya *et al* (28) demonstrated more distinct changes in the HF in patients using propofol versus sevoflurane anesthesia, concluding that sevoflurane has little effect on the cardiac parasympathetic tone. However, in the present study, it was demonstrated that the HRV indices changed with the variations in the stress response, which indicated that remifentanil anesthesia was positively correlated with the stress response. Therefore, if the anesthesia during surgery is too shallow, the body will have strong stress responses to surgical stimuli, thereby causing an increase in the body’s sympathetic nerve excitation and anterior pituitary-adrenal function, and this will therefore alter the body’s endocrine, metabolic and immune functions. These changes lead to a significant increase in a variety of stress response factors, manifesting as high intra-operative levels of COR, glucose and LAC. A corresponding change in HRV also occurs, in which the main factor is an increased LF or LF/HF (31). To further understand the correlation between HRV and the stress response, remifentanil anesthesia was used in OP-CABG in the present study. The two delivery methods, including remifentanil TCI and constant-rate infusion, were used in OP-CABG to compare the changes of indices when the surgical stimulation was large and the hemodynamic indices showed severe changes. There were no significant differences in the intraoperative hemodynamic parameters between the groups, which indicated that the two delivery methods were able to maintain a stable cardiac cycle during surgery (Table II). The release of E and COR in the two groups was effectively inhibited from the time of induction, and the concentrations of epinephrine and cortisol showed no significant fluctuation (Table III). However, the levels of BG and LAC began to increase significantly in the two groups once the sternum had been opened, and from T_5_ the increase was more apparent in group II than in group I, thus indicating that the internal environment more stable in the target-controlled group and showing that the intraoperative catabolism was effectively suppressed in this group (Table III).

As the hemodynamics may be significantly changed by the anastomosis of the circumflex artery, diagonal branch and right coronary artery, the short-term and single-use of α-receptor agonists, such as phenylephrine, was employed. The results demonstrated that more phenylephrine was used in group II than in group I. This may have been due to a clinical requirement against the increased stress that was induced by the accumulation of remifentanil following constant infusion, moving the heart and anastomosing the right coronary and/or circumflex artery. The levels of nitroglycerin and norepinephrine that were used in the two groups were approximately the same (Table I). The majority of the cardiovascular drugs that improve morbidity and mortality, including β-blockers, ACE-inhibitors and statins, also increase HRV. Metoprolol, quinapril, captopril, enalapril and atorvastatin have been shown in a previous study to increase HRV (12). For instance, the clinically observed increase in HRV with β-blockers was likely to be associated with the concomitant beneficial effects on the parasympathetic nervous system and the renin-angiotensin-aldosterone axis (32). However, the amount of metoprolol and esmolol used prior to the surgery was similar in the two groups of the present study. These drugs may have reduced the interference of the intraoperative HRV analysis. Following general anesthesia, the tension of the ANS has been shown to decrease along with the HRV, while the inhibition of the parasympathetic nervous system by propofol becomes stronger, with the main factors of a decreased HF and an increased LF/HF (28). Shinohara *et al* (29) showed that through vagal excitation, remifentanil causes hypotension and bradycardia (decreased LF/HF). In the present study, remifentanil had little effect on the reliability of HRV as an indicator. Therefore, following the induction of general anesthesia, the HRV indices, including TP, LF and HF, began to decrease and differed significantly from the baseline (P<0.05). However, differences in the LF/HF were not significant at T_2_, T_3_, T_6_, T_7_ and T_8_, between groups I and II. When anastomosing the right coronary and/or circumflex arteries (T_5_), the levels of BG and LAC in group II were higher than those in group I. The corresponding TP, LF and LF/HF values in group II were also significantly higher than those in group I at T_5_, thus indicating that sympathetic activity had increased and vagal activity remained inhibited. In addition, a weak correlation between the LF/HF ratio and the plasma level of E, BG and LAC was observed in the two groups at T_5_ and T_7_. This suggests that changes in HRV are correlated with the stress response and may also be correlated with stress-related problems, including heart disease, hypertension, depression and anxiety. Therefore, HRV training may help the individual to better manage stress and anxiety. In the present study, the TCI of remifentanil was used to reduce the imbalance between the sympathetic and vagal nerves, which is conducive to the regulation of the cardiac autonomic nervous system.

The hemodynamic indices did not reveal that the inhibition of the stress response in the target-controlled group was more effective. Following extubation, the HR and MAP and the levels of E and COR increased, while the TP, LF, HF and LF/HF increased significantly. This suggested that despite effective post-operative analgesia, extubation caused strong stimulation, which lead to sympathetic excitation.

In OP-CABG, a TCI of remifentanil is more effective than a constant-rate infusion in the inhibition of the stress response and the maintenance of the cardiac ANS balance. The change in HRV corresponds to the intraoperative stress response and is relevant to the early post-operative recovery of cardiac autonomic function. HRV may act as a key perioperative monitoring index for patients who have undergone OP-CABG.

## Figures and Tables

**Table I.** Comparison of general characteristics between the groups.

Characteristics	Group I (n=32)	Group II (n=33)
Gender (male/female)	25/7	24/9
Age (years)	68.5±10.7	67.9±10.5
Body weight (kg)	68.2±11.1	67.1±10.7
LVEF value (%)	53.9±7.0	54.3±6.9
Remifentanil dosage (mg)	1.68±0.29	1.65±0.28
Metoprolol dosage (mg/day)	55.8±21.2	55.4±20.8
Duration of surgery (min)	239.0±33.2	239.5±33.7
Number of bypasses	3.8±1.1	3.8±1.2
Nitroglycerin dosage (mg)	12.9±2.4	13.3±2.2
Dopamine dosage (mg)	36.4±8.1	35.9±8.8
Norepinephrine dosage (*μ*g)	188.3±20.7	191.4±19.5
Esmolol dosage (mg)	22.4±7.1	23.1±7.6
Phenylephrine dosage (*μ*g)	56.4±8.2	103.8±13.7^a^

a P<0.05 compared with group I (t-test). All data are presented as the mean ± SD with the exception of gender. LVEF, left venticular ejection fraction; SD, standard deviation.

**Table II.** Changes in hemodynamic indices in group I (n=32) and group II (n=33) during the perioperative period at various time points.

Indices	T_0_	T_1_	T_2_	T_3_	T_4_	T_5_	T_6_	T_7_	T_8_
HR (bpm)									
I	56.9±7.4	55.6±6.5	56.8±6.8	58.1±7.0	56.4±6.5	56.0±6.6	56.2±7.0	69.8±9.5^a^	58.2±6.8
II	56.8±7.5	55.6±6.8	56.9±7.0	57.9±6.8	56.7±6.9	56.2±7.0	56.4±7.1	69.8±9.7^a^	58.7±7.3
MAP (mmHg)									
I	72.3±11.6	69.9±10.2	71.1±11.2	72.2±10.4	71.5±9.9	69.3±10.7	72.1±10.2	82.1±10.8^a^	74.1±10.5
II	72.6±12.0	69.7±10.1	71.3±10.7	72.5±10.3	72.0±9.7	69.7±10.1	71.6±10.5	86.2±10.9^a^	74.1±10.5
CVP (mmHg)									
I	4.7±0.8	4.8±0.9	4.8±0.6	4.8±0.5	5.0±0.8	5.1±0.9	5.1±0.6	5.1±0.7	5.0±0.9
II	4.7±1.0	4.8±0.8	4.9±0.9	4.8±0.6	5.0±0.6	5.2±0.8	5.0±0.8	5.2±1.1	5.1±0.9
PAP (mmHg)									
I	18.6±5.1	19.1±4.9	18.9±4.4	18.9±4.8	19.3±4.9	19.5±5.1	18.9±5.1	19.1±5.0	18.8±5.0
II	18.7±4.9	19.1±5.0	18.8±4.8	19.0±4.7	19.4±4.9	19.4±5.1	19.2±5.0	19.3±5.2	18.7±5.1
PCWP (mmHg)									
I	10.8±2.2	10.7±2.1	10.9±2.2	11.0±1.8	10.7±1.9	11.1±2.1	11.0±1.8	10.7±1.9	10.8±2.1
II	10.7±2.1	10.7±2.2	10.9±2.3	11.0±1.9	10.8±2.1	10.9±2.1	11.0±1.8	10.8±1.9	10.9±2.2

a P<0.05 compared with T_0_ (t-test). All data are presented as the mean ± SD. HR, heart rate; MAP, mean arterial pressure; CVP, central venous pressure; PAP, pulmonary arterial pressure; PCWP, pulmonary capillary wedge pressure; SD, standard deviation.

**Table III.** Changes in the plasma levels of E, COR, BG and LAC in group I (n=32) and group II (n=33) at various time points.

Indices	T_0_	T_2_	T_3_	T_5_	T_6_	T_7_	T_8_
E (pg/ml)							
I	105±25	108±29	107±22	102±22	108±29	318±86^a^	108±25
II	106±23	110±27	104±30	101±24	104±20	327±74^a^	109±27
COR (ng/ml)							
I	76.6±15.8	74.2±14.9	79.8±10.6	75.8±16.5	75.8±15.8	98.1±20.9^a^	80.1±16.6
II	78.3±16.1	74.8±15.1	79.9±10.9	76.5±15.9	76.0±14.9	99.2±21.8^a^	80.3±17.8
BG (mmol/l)							
I	5.3±0.8	5.1±0.9	7.1±1.1^a^	7.3±1.4^a^	7.4±1.9^a^	7.5±1.1^a^	5.6±0.9
II	5.5±0.9	5.6±0.7	7.4±1.2^a^	9.4±2.5^ab^	10.4±1.8^ab^	10.9±2.1^ab^	5.9±1.0
LAC (mmol/l)							
I	0.9±0.3	1.0±0.3	1.7±0.5^a^	1.8±0.4^a^	1.7±0.2^a^	1.6±0.3^a^	1.0±0.4
II	1.0±0.3	1.0±0.4	1.8±0.6^a^	2.9±0.3^ab^	2.4±0.2^ab^	2.2±0.3^ab^	1.0±0.4

a P<0.05 compared with T_0_;

b P<0.05 compared with Group I. All data are presented as the mean ± SD. E, epinephrine; COR, cortisol; BG, blood glucose; LAC, lactate; SD, standard deviation.

**Table IV.** Changes in HRV in group I (n=32) and group II (n=33) at various time points.

Indices	T_0_	T_2_	T_3_	T_5_	T_6_	T_7_	T_8_
TP (msec^2^/Hz)							
I	3.25±0.41	2.48±0.29^a^	2.45±0.61^a^	4.55±0.52^a^	2.65±0.31^a^	5.75±0.82^a^	2.35±0.32^a^
II	3.29±0.39	2.43±0.31^a^	2.55±0.67^a^	5.85±0.71^ab^	2.85±0.34^a^	5.80±0.90^a^	2.39±0.37^a^
LF (msec^2^/Hz)							
I	2.65±0.33	1.94±0.35^a^	1.86±0.41^a^	3.96±0.73^a^	2.03±0.38^a^	5.09±0.93^a^	1.72±0.63^a^
II	2.67±0.34	1.96±0.33^a^	1.87±0.39^a^	5.09±0.79^ab^	2.06±0.39^a^	5.13±0.89^a^	1.75±0.60^a^
HF (msec^2^/Hz)							
I	2.29±0.25	1.57±0.29^a^	1.54±0.25^a^	1.50±0.35^a^	1.51±0.37^a^	1.72±0.31^a^	1.64±0.24^a^
II	2.31±0.29	1.59±0.26^a^	1.53±0.23^a^	1.51±0.32^a^	1.50±0.33^a^	1.68±0.35^a^	1.62±0.27^a^
LF/HF							
I	1.79±0.86	1.26±0.52^a^	1.22±0.36^a^	2.75±0.96^a^	1.28±0.56^a^	3.36±0.86^a^	1.39±0.56^a^
II	1.78±0.84	1.28±0.56^a^	1.23±0.40^a^	3.86±1.16^ab^	1.29±0.56^a^	3.40±0.91^a^	1.43±0.61^a^

a P<0.05 compared with T_0_;

b P<0.05 compared with Group I. All data are presented as the mean ± SD. HRV, heart rate variability; SD; standard deviation; TP, total power; LF, low frequency power; HF, high frequency power; LF/HF, LF/HF power ratio.
